# Clinical characteristics and genetic analysis of pediatric patients with sodium channel gene mutation-related childhood epilepsy: a review of 94 patients

**DOI:** 10.3389/fneur.2023.1310419

**Published:** 2023-12-18

**Authors:** Hongjun Fang, Wenjing Hu, Qingyun Kang, Xiaojun Kuang, Lijuan Wang, Xiao Zhang, Hongmei Liao, Liming Yang, Haiyan Yang, Zhi Jiang, Liwen Wu

**Affiliations:** Neurology Department, Hunan Children's Hospital, Changsha, China

**Keywords:** epilepsy, sodium channel, gene, mutation, pediatrics

## Abstract

**Objective:**

This study aimed to examine the clinical and gene-mutation characteristics of pediatric patients with sodium channel gene mutation-related childhood epilepsy and to provide a basis for precision treatment and genetic counseling.

**Methods:**

The clinical data from 94 patients with sodium channel gene mutation-related childhood epilepsy who were treated at Hunan Children's Hospital from August 2012 to December 2022 were retrospectively evaluated, and the clinical characteristics, gene variants, treatment, and follow-up status were analyzed and summarized.

**Results:**

Our 94 pediatric patients with sodium channel gene variant-related childhood epilepsy comprised 37 girls and 57 boys. The age of disease onset ranged from 1 day to 3 years. We observed seven different sodium channel gene variants, and 55, 14, 9, 6, 6, 2, and 2 patients had *SCNlA, SCN2A, SCN8A, SCN9A, SCN1B, SCN11A*, and *SCN3A* variants, respectively. We noted that 52 were reported variants and 42 were novel variants. Among all gene types, *SCN1A, SCN2A*, and *SCN8A* variants were associated with an earlier disease onset age. With the exception of the *SCN1B*, the other six genes were associated with clustering seizures. Except for variants *SCN3A* and *SCN11A*, some patients with other variants had status epilepticus (SE). The main diagnosis of children with *SCN1A* variants was Dravet syndrome (DS) (72.7%), whereas patients with *SCN2A* and *SCN8A* variants were mainly diagnosed with various types of epileptic encephalopathy, accounting for 85.7% (12 of 14) and 88.9% (8 of 9) respectively. A total of five cases of sudden unexpected death in epilepsy (SUDEP) occurred in patients with *SCN1A, SCN2A*, and *SCN8A* variants. The proportion of benign epilepsy in patients with *SCN9A, SCN11A*, and *SCN1B* variants was relatively high, and the epilepsy control rate was higher than the rate of other variant types.

**Conclusion:**

Sodium channel gene variants involve different epileptic syndromes, and the treatment responses also vary. We herein reported 42 novel variants, and we are also the first ever to report two patients with *SCN11A* variants, thereby increasing the gene spectrum and phenotypic profile of sodium channel dysfunction. We provide a basis for precision treatment and prognostic assessment.

## 1 Introduction

Epilepsy is a common neurologic disorder that reflects diverse causes, including genetic factors, brain disease, and generalized or systemic disease. As our knowledge of genetics gradually improves, we now recognize an abundance of findings that show that ion channel gene mutations are intimately associated with epilepsy (1). Among these mutations, those encoding for sodium channels are highly correlated with childhood epilepsy (2). The voltage-gated sodium channel (Nav) is a protein complex consisting of one α subunit and two β subunits and is primarily present in the central nervous system (CNS), peripheral nervous system (PNS), skeletal muscles, and myocardium (3). The α subunit of the Nav channel is a functional subunit of ~2,000 amino acids that is highly conserved. There are nine different Nav α subtypes (Nav1.1–Nav1.9), and mammalian tissues express all nine (4). These subtype proteins are named Nav1.1–Nav1.9 and the corresponding genes are designated *SCN1A*–*SCN5A* and *SCN8A*–*SCN11A*. Of these, Nav1.1, Nav1.2, Nav1.3, and Nav1.6 are highly expressed in the central nervous system (CNS) and two different subtypes are expressed in muscles (Nav1.4 in adult skeletal muscle and Nav1.5 in myocardium), while Nav1.7, Nav1.8, and Nav1.9 are principally expressed in the peripheral nervous system (5). There are four Nav channel β subunits that are encoded by four genes, i.e., *SCN1B*–*SCN4B* (6). Nav channel mutations are related to epilepsy, including α subunits, such as Nav1.1 (*SCN1A*), Nav1.2 (*SCN2A*), Nav1.3 (*SCN3A*), Nav1.6 (*SCN8A*), and Nav1.7 (*SCN9A*), and the β subunit (*SCN1B*) (7). In order to strengthen our understanding of the different sodium channel genotypes and clinical phenotypes, and to guide personalized treatment, determination of disease prognosis, and genetic counseling, we herein summarized the clinical and gene-mutation characteristics of 94 sodium channel gene mutation-related childhood epilepsy patients who were treated at Hunan Children's Hospital. This review, therefore, comprises the broadest analysis of sodium channel genes currently available.

## 2 Participants and methods

### 2.1 Participants

This study was a retrospective summary of cases. We collected data from epileptic patients who were treated in the neurology outpatient clinic or the inpatient unit at Hunan Children's Hospital from August 2012 to December 2022, and who harbored sodium channel gene variants upon genetic testing. These patients sought medical attention for epilepsy of unknown cause and underwent whole-exome sequencing for personalized treatment, prognostic assessment, and genetic counseling. A detailed clinical data registry (including name, sex, date of birth, age at disease onset, status of mental development, seizure presentation, perinatal condition, past medical history, family history, response to anticonvulsants, genetic test results, head MRI, and electroencephalography) was established for every patient. Patients were followed up through outpatient visits, readmission, or telephone follow-ups. The clinical and genetic characteristics of these patients were retrospectively analyzed, and the guardians of all pediatric patients provided signed informed consent. The clinical data of patients and the DNA from the peripheral blood of the patients and their parents were also collected. This study was approved by the Ethics Committee of Hunan Children's Hospital.

### 2.2 Methods

Agilent exon chip capture + high-throughput sequencing was used for whole-exome sequencing to screen for gene mutations in pediatric patients, and first-generation sequencing was executed on the patients and their parents to validate mutation sites and determine the source of the mutation.

The response to anticonvulsants was divided into four types: controlled (no seizure for at least 3 months), reduced (seizure frequency decreased by ≥25%), ineffective (seizure frequency decreased by <25%), and worsened. Controlled and reduced responses were considered to be effective responses (8).

All children were scored for intellectual development: children aged 6 years were evaluated for intelligence using the Chinese standardized Gesell scale; children older than 6 years were assessed for intelligence using the Wechsler Intelligence Scale for Children. A score of <70 indicates intellectual disability; 50–69 indicates mild intellectual disability; 35–49 indicates moderate intellectual disability; 20–34 indicates severe intellectual disability; and <20 indicates extremely severe intellectual disability (9, 10).

### 2.3 Statistical analysis

We implemented the SPSS24.0 statistics suite for this analysis. Measurement data with a normal distribution are presented as the mean ± standard deviation, and measurement data with a non-normal distribution are presented as the median. In addition, qualitative data are presented as the number of patients (percentage). The independent-sample Kruskal–Wallis test was applied to compare the differences in age at onset with respect to the seven gene variants, and we conducted pairwise comparisons. The Mann–Whitney *U*-test was used to compare differences in the responses to these seven mutated genes.

## 3 Results

### 3.1 Patient demographics

**Figure 1:**
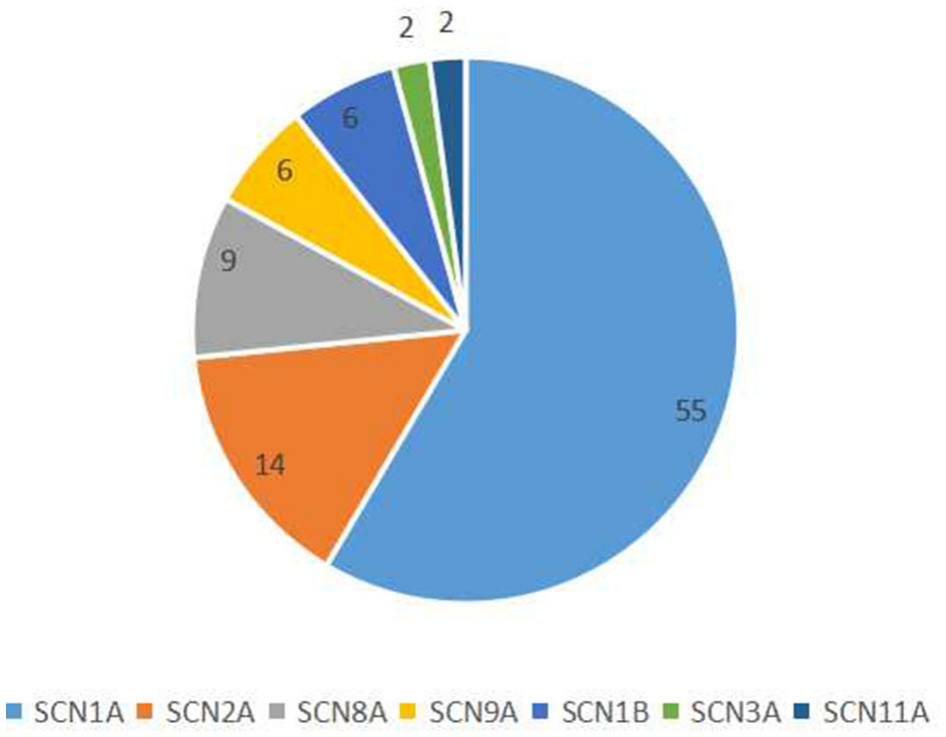
Classification of sodium channel gene variants.

We collected data from 94 patients with sodium channel gene variant-related childhood epilepsy, comprising 37 girls and 57 boys (see Table 1 for clinical presentations and gene-variant results). The age at onset was 1 day to 3 years, and the median age was 7.0 (4.5, 10.2) months. The children's parents were not consanguineous and the patients were not related to each other. We detected a total of seven different sodium channel gene variants in our patients, and 55, 14, 9, 6, 6, 2, and 2 patients had *SCNlA, SCN2A, SCN8A, SCN9A, SCN1B, SCN11A*, and *SCN3A* variants, respectively (Figure 1). A total of 62 patients harbored missense variants; 11 were frameshift variants, 10 were splicing variants, nine were non-sense variants, 1 was a deletion variant, and 1 was a chimeric duplication variant. A total of 52 cases were reported to be pathogenic variants, 42 were novel variants, 22 were inherited variants, and 72 were *de novo* variants. There were more male patients with sodium channel-related epilepsy than female patients. The most common variant was *SCN1A*, accounting for 58.5% of cases, and missense variants constituted the most common type, accounting for 66.0% of cases. Pediatric patients mainly had *de novo* variants (accounting for 76.6%) and some inherited-mutation patients presented with genetic epilepsy with febrile seizures plus (GEFS+) and febrile seizures plus (FS+) that showed good prognoses (Table 2).

**Table 1:** Clinical findings of novel and reported sodium channel gene variants.

**Pt**	**Gene type**	**Sex**	**Age of onset**	**Seizure types**	**SE**	**Diagnosis**	**Development**	**Variant**	**Variant type**	**Inheritance**	**Novel/ reported**	**Age of last follow-up**	**Response toASMs**	**ASMs**		
														**Controlled**	**Reduction**	**Failure**
1	SCN1A	F	10 m	FS, GTCS	N	DS	Severe delay	c.4314_4315insGATGGATATAATA (p.Tyr1439Aspfs^*^8)	Frameshift	*De novo*	Novel	9 y 1 m	Controlled	TPM	VPA	
2	SCN1A	F	6 m	GTCS, FS	N	DS	Severe delay	c.2450T>C (p.Leu817Pro)	Missense	*De novo*	Novel	5 y 4 m	Reduction		VPA, CLB	
3	SCN1A	F	1 y 8 m	GTCS	N	CFS	Normal	c.2830G>A (p.Val944Met)	Missense	*De novo*	Novel	7 y 9 m	Controlled	LEV		
4	SCN1A	M	10 m	GTCS, Myc, FS	N	DS	Severe delay	c.4261A>G (p.K1421E)	Missense	*De novo*	Novel	10 y 4 m	Reduction		VPA	OXC, LEV
5	SCN1A	F	8 m	GTCS, FS, Myc	Y	DS	Severe delay	c.2693delA (p.Asp898ValfsTer9)	Frameshift	Maternal	Novel	9 y 6 m	Reduction		TPM, CLB	LEV, VPA
6	SCN1A	F	2 y	FS	Y	EE	Severe delay	c. 4338+8(IVS24) T>C	Splicing	*De novo*	Novel	10 y	Dead			VPA, TPM, LEV
7	SCN1A	F	6 m	GTCS	N	GEFS+	Normal	c.5416G>A (p.E1806K)	Missense	Paternal	Novel	6 y 2 m	Reduction		PB	
8	SCN1A	F	10 m	GTCS	N	FS+	Normal	c.337C>G (p.P113A)	Missense	*De novo*	Novel	6 y 7 m	Controlled	VPA, LEV		
9	SCN1A	M	6 m	FS, GTCS	N	DS	Moderate delay	c.965-1G>T	Splicing	*De novo*	Novel	4 y 4 m	Reduction		VPA, TPM	LEV
10	SCN1A	M	6 m	FS	Y	DS	Moderate delay	c.2803C>T (p.R935C)	Missense	*De novo*	Novel	6 y 7 m	Reduction		VPA	LEV, TPM
11	SCN1A	M	5 m	FS	Y	DS	Severe delay	c.2804G>A (p.R935H)	Missense	*De novo*	Novel	8 y	Reduction		CLB	KD, VPA, TPM
12	SCN1A	M	4 m	FS	Y	DS	Severe delay	c.1312G>T (p.E438^*^)	Non-sense	*De novo*	Novel	5 y	Reduction		KD	LEV, VPA, TPM
13	SCN1A	M	4 m	FS	Y	DS	Moderate delay	c.1888delC (p.R630fs)	Frameshift	*De novo*	Novel	6 y 9 m	Reduction		VPA, TPM	
14	SCN1A	F	5 m	FS, GTCS	N	DS	Moderate delay	c.5666T>A (p.M1889K)	Missense	*De novo*	Novel	7 y 4 m	Controlled	VPA		
15	SCN1A	M	17 m	FS	N	GEFS+	Normal	c.3569A>T (p.K1190M)	Missense	Paternal	Novel	6 y 7 m	Controlled	VPA		
16	SCN1A	M	5 m	FS	N	DS	Moderate delay	c.3096A>T (p.E1032D)	Missense	*De novo*	Novel	4 y	Reduction		VPA, TPM	LEV
17	SCN1A	M	2 m	FS	N	DS	Severe delay	c.4224delG (p.Trp1408^*^)	Non-sense	*De novo*	Novel	3 y 10 m	Controlled	CLB	TPM	VPA
18	SCN1A	M	10 m	FS	N	DS	Moderate delay	c.4271C>T (p.Ser1424Phe)	Missense	*De novo*	Novel	5 y 1 m	Reduction		VPA, TPM	
19	SCN1A	F	9 m	FS	Y	DS	Moderate delay	c.1084T>G (p.Tyr362Asp)	Missense	*De novo*	Novel	3 y 4 m	Failure			VPA, TPM
20	SCN1A	M	7 m	GTCS	N	DS	Mild delay	c.1996C>T (p.Gln666^*^)	Non-sense	*De novo*	Novel	10 y	Reduction		VPA, KD	LEV
21	SCN1A	F	8 m	GTCS	N	GEFS+	Normal	c.3520T>C (p.C1174R)	Missense	Paternal	Novel	6 y	Controlled	LEV		
22	SCN1A	M	8 m	GTCS, FS	Y	DS	Severe delay	c.4180A>G (p.T1394A)	Missense	*De novo*	Novel	3 y 9 m	Failure			VPA, TPM, CLB
23	SCN1A	M	7.5 m	GTCS, FS, Myc	Y	DS	Severe delay	c.3304delT (p.Y1102Tfs^*^5)	Frameshift	*De novo*	Novel	5 y 7 m	Reduction		VPA, TPM	LEV
24	SCN1A	M	3 m	SS	N	WS	Severe delay	c.4285-3T>C	Splicing	Maternal	Novel	5 y 6 m	Controlled	ACTH, TPM		
25	SCN1A	M	6 m	GTCS	Y	FS+	Mild delay	c.4980delC (p.L1660Lfs^*^4)	Frameshift	*De novo*	Novel	13 y	Controlled	LEV, DZP		
26	SCN1A	M	7 m	GTCS	N	CFS	Mild delay	c.4970G>C (p.R1657P)	Missense	*De novo*	Novel	4 y	Reduction		VPA	
27	SCN1A	F	5 m	GTCS, FS	Y	DS	Severe delay	C.5530_5533del (p.Pro1844Thrfs^*^13)	Frameshift	*De novo*	Novel	2 y 5 m	Reduction		VPA, TPM	LEV
28	SCN1A	M	5.5 m	GTCS	N	DS	Moderate delay	Exon(16-21)dup	Repeat	*De novo*	Novel	3 y 9 m	Failure			VPA, LEV
29	SCN1A	M	8 m	GTCS, Myc, AAS, FS	N	DS	Moderate delay	c.5484dupA (P.S1829Ifs^*^8)	Frameshift	*De novo*	Novel	10 y 1 m	Reduction		CLB	VPA, TPM, LEV, OXC, LCS, CZP
30	SCN2A	M	1 y 11 m	FS	N	EE	Moderate delay	c.1383+2T>G (splice-5)	Splicing	*De novo*	Novel	5 y 3 m	Controlled	LEV	PB	
31	SCN2A	F	2 m	FS	Y	EE	Severe delay	Lossel Exon:3-28	Deletion	*De novo*	Novel	1 y 8 m	Reduction		CLB, LCS	LEV
32	SCN8A	M	7 m	FS	N	BFE	Normal	c.4642A>C (p.Lys1548Gln)	Missense	Maternal	Novel	4 y 2 m	Controlled	VPA		
33	SCN9A	M	5 m	FS	N	EE	Severe delay	c.5847T>G (p.S1949R)	Missense	*De novo*	Novel	9 y	Controlled	VPA		
34	SCN9A	M	3 y	GTCS	N	GEFS+	Normal	c.2958G>T (p.K986N)	Missense	Paternal	Novel	13 y	Controlled	VPA, LEV		
35	SCN9A	M	9 m	GTCS	N	GEFS+	Normal	c.41A>G (p.H14R)	Missense	Maternal	Novel	6 y 10 m	Controlled	VPA		
36	SCN9A	M	1 y	GTCS	N	GEFS+	Normal	c.5471G>A (p.R1824Q)	Missense	Paternal	Novel	14 y	Controlled	VPA		
37	SCN9A	M	10 m	GTCS	N	CFS	Normal	c.1889T>G (p.Leu630Arg)	Missense	Maternal	Novel	6 y	Controlled	VPA		
38	SCN9A	F	8 m	GTCS	N	GEFS+	Mild delay	c.1286G>T (p.Arg429Leu)	Missense	Maternal	Novel	4 y	Controlled	VPA		LEV
39	SCN1B	F	6 m	FS	Y	EE	Moderate delay	c.667C>G (p.H223D)	Missense	*De novo*	Novel	6 y	Failure			VPA
40	SCN1B	F	8 m	GTCS	N	GEFS+	Normal	c.785G>A (p.C262Y)	Missense	Maternal	Novel	14 y 4 m	Controlled	VPA		
41	SCN3A	M	2 y 7 m	FS, SS	N	EE, autism	Severe delay	c.634G>A (p.Val212Ile)	Missense	*De novo*	Novel	6 y 2 m	Reduction		VPA	OXC
42	SCN11A	M	1 y 1 m	FS	Y	EE	Moderate delay	c.1646dupA (p.N549fs)	Frameshift	Maternal	Novel	11 y	Controlled	VPA		OXC, PB
43	SCN1A	M	7 m	FS, GTCS	Y	DS	Severe delay	c.602+1G>A	Splicing	*De novo*	Reported	4 y 3 m	Reduction		CLB, KD	VPA, TPM
44	SCN1A	M	6 m	GTCS	N	EE	Severe delay	c.1009G>A (p.Gly337Arg)	Missense	*De novo*	Reported	6 y	Reduction		VPA	LTG, LEV
45	SCN1A	M	2y	GTCS, FS	N	EE	Severe delay	c.2069G>C (p.Arg690Thr)	Missense	*De novo*	Reported	6 y 6 m	Reduction		VPA	
46	SCN1A	M	3 m	FS	N	EE	Mild delay	c.2416-2A>G	Splicing	*De novo*	Reported	4 y 1 m	Reduction		VPA, CLB	
47	SCN1A	F	4 m	FS	Y	DS	Severe delay	c.4853-2A>C	Splicing	Maternal	Reported	4 y	Reduction		TPM, VPA, CZP	LEV
48	SCN1A	M	7 m	FS	Y	DS	Severe delay	c.4973C>A (p.T1658K)	Missense	*De novo*	Reported	6 y	Reduction		CLB	VPA, CZP
49	SCN1A	F	4 m	FS, GTCS	Y	DS	Severe delay	c.1028+2T>C	Splicing	*De novo*	Reported	8 y 7 m	Dead			VPA, LEV
50	SCN1A	F	7 m	FS	Y	DS	Mild delay	c.2177-6A>G	Splicing	*De novo*	Reported	7 y 4 m	Controlled	KD	VPA	LEV
51	SCN1A	M	9 m	FS	Y	DS	Severe delay	c.2782C>T (p.Q928^*^)	Non-sense	*De novo*	Reported	9 y	Reduction		KD	LEV, VPA, TPM
52	SCN1A	M	4 m	FS	Y	DS	Moderate delay	c.4359T>G (p.Y1453^*^)	Non-sense	*De novo*	Reported	4 y 8 m	Reduction		VPA, CLB	LEV
53	SCN1A	M	5 m	FS	N	DS	Moderate delay	c.301C>T (p.R101W)	Missense	*De novo*	Reported	6 y 3 m	Controlled	CLB	TPM	VPA
54	SCN1A	M	4 m	FS, Myc	N	DS	Severe delay	c.1363C>T (p.Q455^*^)	Non-sense	*De novo*	Reported	4 y 6 m	Reduction		CLB	VPA, LEV
55	SCN1A	M	8 m	FS	N	DS	Severe delay	c.3705+5G>A	Splicing	*De novo*	Reported	11 y 5 m	Reduction		CLB, KD	VPA, LEV, TPM
56	SCN1A	F	5 m	FS	Y	DS	Severe delay	c.2350_2351delTT (p.F784Hfs^*^14)	Splicing	*De novo*	Reported	5 y 9 m	Reduction		CLB, VPA	LEV, TPM
57	SCN1A	F	10 m	FS, GTCS	N	CFS	Normal	c.4112G>C (p.G1371A)	Missense	*De novo*	Reported	5 y	Controlled	VPA, TPM, CZP		
58	SCN1A	F	7 m	FS	Y	DS	Severe delay	c.1596delA (p.G533Vfs^*^11)	Frameshift	*De novo*	Reported	4 y 9 m	Failure			VPA, LEV, TPM, CZP
59	SCN1A	M	3 m	FS	Y	DS	Severe delay	c.5010_c.5013delGTTT (p.L1670Lfs^*^9)	Frameshift	*De novo*	Reported	6 y 11 m	Reduction		VPA, TPM	
60	SCN1A	M	5 m	GTCS	Y	DS	Moderate delay	c.301C>T (p.R101Wrs)	Missense	*De novo*	Reported	6 y 3 m	Reduction		VPA, LEV	
61	SCN1A	M	9 m	GTCS, FS, Myc	N	DS	Severe delay	c.4926G>C (p.Arg1642Ser)	Missense	*De novo*	Reported	3 y 7 m	Reduction		VPA, CZP	
62	SCN1A	M	11 m	GTCS	Y	DS	Severe delay	c.302G>A (p.Arg101Gln)	Missense	*De novo*	Reported	9 y	Controlled	VPA, TPM, LEV		
63	SCN1A	F	4 m	FS	Y	DS	Moderate delay	c.4168G>A (p.V1390M)	Missense	*De novo*	Reported	3 y 9 m	Reduction		VPA, TPM	OXC
64	SCN1A	M	8 m	FS	Y	DS	Severe delay	c.1624C>T (p.R542^*^)	Non-sense	*De novo*	Reported	6 y 3 m	Reduction		CLB	LEV, VPA
65	SCN1A	M	2 y	GTCS, FS, AT	N	EE	Moderate delay	c.1719C>A (p.S573R)	Missense	*De novo*	Reported	13 y	Reduction		LEV, VPA	OXC
66	SCN1A	M	6 m	FS, Myc	N	DS	Severe delay	c.2214G>A (p.Trp738^*^)	Non-sense	*De novo*	Reported	3 y 7 m	Reduction		VPA, CLB	
67	SCN1A	M	11 m	FS	N	GEFS+	Normal	c.2462C>T (p.Ala821Val)	Missense	Maternal	Reported	7 y 7 m	Controlled	LCS		
68	SCN1A	F	7 m	FS	Y	DS	Severe delay	C.2624C>G (P.Thr875Arg)	Missense	*De novo*	Reported	3 y	Reduction		VPA	
69	SCN2A	F	4 m	SS, FS	N	WEST	Severe delay	c.3454G>A (p.Ala1152Thr)	Missense	*De novo*	Reported	6 y 10 m	Controlled	CLB		ACTH, VPA, LEV
70	SCN2A	F	3 y	FS	N	EE, autism	Severe delay	c.5236T>C (p.C1746R)	Missense	*De novo*	Reported	5 y 10 m	Controlled	OXC		LEV, VPA
71	SCN2A	M	3 m	FS	Y	EE	Severe delay	c.1837G>A (p.Val613Met)	Missense	*De novo*	Reported	3 y 3 m	Reduction		VPA	LEV, TPM, OXC
72	SCN2A	F	2 d	FS, SS	N	OS	Severe delay	c.4959G>C (p.L1653F)	Missense	*De novo*	Reported	7 m	Dead			ACTH, PB, LEV
73	SCN2A	F	2 d	FS, SS	N	OS-WS-LGS	Severe delay	c.4498G>A (p.A1500T)	Missense	*De novo*	Reported	5 y 2 m	Failure			VPA, TPM, LEV, PB
74	SCN2A	F	1 d	FS	N	EE	Moderate delay	c.605C>T (p.A202V)	Missense	*De novo*	Reported	4 y	Controlled	OXC		LEV, PB
75	SCN2A	M	2 y 1 m	FS	N	EE	Severe delay	c.304C>T (p.R102X)	Non-sense	*De novo*	Reported	7 y 5 m	Reduction		VPA	LEV
76	SCN2A	M	1 y 11 m	FS, TODD	Y	BFE	Normal	c.1904T>C (p.Leu635Pro)	Missense	Paternal	Reported	5 y 1 m	Controlled	LEV		
77	SCN2A	F	8 m	FS	N	BFE	Normal	c.5999T>C (p.Ile2000Thr)	Missense	Maternal	Reported	6 y 3 m	Controlled	VPA		
78	SCN2A	F	2 m	FS	N	EE	Severe delay	c.3973G>A (p.Val1325Ile)	Missense	*De novo*	Reported	5 y	Reduction		OXC	LEV, TPM, PB
79	SCN2A	M	3 m	FS	N	EE	Moderate delay	c.5668A>G (p.K1890E)	Missense	*De novo*	Reported	1 y 9 m	Controlled	OXC		LEV
80	SCN2A	F	1 d	FS	N	EIMFS	Severe delay	C.1285G>A (P.Glu429Lys)	Missense	*De novo*	Reported	1 y 9 m	Controlled	LCS		VPA, LEV, OXC
81	SCN8A	F	3 m	FS	N	EE	Severe delay	c.1099A>G( p.Met367Val)	Missense	*De novo*	Reported	11 y 5 m	Controlled	CBZ	VPA	
82	SCN8A	M	8m	FS	N	EE	Moderate delay	c.4771G>A (p.Val1591Met)	Missense	Paternal	Reported	8 y 5 m	Controlled	OXC		VPA, TPM
83	SCN8A	M	4 m	FS	N	EE	Severe delay	c.2549G>A (p.R850Q)	Missense	*De novo*	Reported	5 y 3 m	Dead			VPA, TPM, LEV
84	SCN8A	M	5 m	FS	N	EE	Severe delay	c.4423G>A (p.G1475R)	Missense	*De novo*	Reported	3 y 3 m	Controlled	OXC	VPA	LEV, PB
85	SCN8A	M	1 m	FS, SS	N	WS	Severe delay	c. 2300C > T P.T767I	Missense	*De novo*	Reported	6 y 7 m	Dead			VPA, TPM, PB, KD
86	SCN8A	M	1 y 3 m	FS, SS, TS, AAS	N	LGS	Severe delay	c.5479A>G (p.(Ile1827Val))	Missense	*De novo*	Reported	7 y 2 m	Failure		VGB, KD	ACTH, LEV, OXC, CLB
87	SCN8A	F	6 m	FS	Y	EE	Severe delay	c.2945C>T (p.A982V)	Missense	*De novo*	Reported	6 y 8 m	Reduction		OXC, LCS	VPA, TPM, LEV
88	SCN8A	F	5 m	FS	Y	EE	Severe delay	c.5630A>G (p.N1877S)	Missense	*De novo*	Reported	11 m	Reduction		OXC	LEV
89	SCN1B	F	1 y 2 m	GTCS	N	GEFS+	Moderate delay	c.460A>G (p.Met154Val)	Missense	Maternal	Reported	10 y 5 m	Controlled			
90	SCN1B	F	1 y 10 m	GTCS	N	GEFS+	Normal	c.133C>T (p.Arg45Cys)	Missense	Maternal	Reported	8 y 7 m	Controlled	VPA		
91	SCN1B	F	1 y 5 m	GTCS, FS	N	EE	Moderate delay	c.38T>C (p.Leu13Pro)	Missense	*De novo*	Reported	8 y 5 m	Controlled	LCS	VPA, TPM	LEV
92	SCN1B	M	1 y 10 m	FS	N	EE	Severe delay	c.287G>A (p.Arg96Gln)	Missense	*De novo*	Reported	6 y	Controlled	DZP		
93	SCN3A	M	2 y 1 m	FS	N	EP	Mild delay	c.3068G>A (p.R1023Q)	Missense	Paternal	Reported	7 y 9 m	Reduction		VPA	
94	SCN11A	F	10 m	GTCS	N	GEFS+	Normal	c.2011T>C (p.S671P)	Missense	Paternal	Reported	14 y	Controlled	VPA, LEV		

ASMs, antiseizure medications; FS+, febrile seizure plus; DS, Dravet syndrome; WS, West syndrome; CFS, complex febrile seizure; EE, unclassified epileptic encephalopathy; BF(I)E, benign familial (infantile) epilepsy; SE, status epilepticus; GEFS+, genetic epilepsy with febrile seizures plus; EIMFS, epilepsy of infancy with migrating focal seizures; EP, unclassified epilepsy; VPA, valproate; LEV, levetiracetam; OXC, oxcarbazepine; TPM, topiramate; KD, ketogenic diet; LCS, lacosamide; PB, phenobarbital; CLB, clobazam; CZP, clonazepam; ACTH, adrenocorticotropic hormone; DZP, diazepam; FS, focal seizure; SS, spasm; Myc, myoclonic; AAS, atypical absence seizure; GTCS, generalized tonic-clonic seizure; AT, atonic seizure; TS, tonic seizure; TODD, Todd's paralysis; y, year; m, month; N, no; Y, yes; F, female; M, male.

**Table 2:** Distributional characteristics of sodium channel gene variants.

	**Total (*n* = 94)**	**SCN1A (*n* = 55)**	**SCN2A (*n* = 14)**	**SCN8A (*n* = 9)**	**SCN9A (*n* = 6)**	**SCN1B (*n* = 6)**	**SCN3A (*n* = 2)**	**SCN11A (*n* = 2)**
Missense variant	62	27	11	9	6	6	2	1
Non-sense variant	9	8	1	0	0	0	0	0
Frameshift variant	11	10	0	0	0	0	0	1
Splicing variant	10	9	1	0	0	0	0	0
Deletion	1	0	1	0	0	0	0	0
Repeat	1	1	0	0	0	0	0	0
*De novo* variant	72	48	12	7	1	3	1	0
Inherited gene variant	22	7	2	2	5	3	1	2
Novel variant	42	29	2	1	6	2	1	1

### 3.2 Clinical presentation of sodium channel-related epilepsy patients

Out of the seven genes we evaluated, the genotypes with early disease onset were *SCN1A, SCN2A*, and *SCN8A*, and the median age at onset was 7.0 (5.0, 9.0), 3.0 (0.1, 23.0), and 5.0 (3.5, 7.5) months, respectively. The age at onset for *SCN1A* ranged from 2 months to 2 years. There was one patient with disease onset within 3 months, 19 patients within 3–6 months, 31 patients between 6 and 12 months, and five patients manifested the disease onset after 1 year of age. The age at onset of patients with the *SCN2A* ranged from day 1 of birth to 3 years of age. There were eight patients with disease onset within 3 months (accounting for 57.1%), of whom four exhibited disease onset within 1–2 days after birth. There were six patients with disease onset after 3 months (accounting for 42.9%), of whom four patients developed the disease after 1 year of age. The age at disease onset of patients with the *SCN8A* ranged from 1 month to 1 year and 3 months, of whom one patient showed disease onset within 3 months (accounting for 11.1%), and eight patients after 3 months (accounting for 88.9%), of whom one patient developed the disease after 1 year of age. Disease onset was later for *SCN9A, SCN1B, SCN3A*, and *SCN11A* variant patients. With the exception of the *SCN1B*, the other six genes were associated with clustering seizures; these were ranked in descending order of patient percentage as *SCN1A* (78.2%), *SCN2A* (64.3%), *SCN8A* (55.6%), *SCN3A* (50%), *SCN11A* (50%), and *SCN9A* (33.3%). With the exception of the *SCN3A*, the genes had a tendency to correlate with fever sensitivity. Fever sensitivity occurred in all patients with the *SCN9A, SCN11A*, and *SCN1B* variants, while the proportions of individuals with *SCN1A, SCN2A*, and *SCN8A* variants who developed fever sensitivity were 92.7, 21.4, and 11.1%, respectively. With the exception of *SCN3A* and *SCN11A* variants, several patients with the other gene variants experienced SE, and the SE duration was the longest in *SCN1A* variant patients, ranging from 20 min to 4 h. There were varying proportions of the seven gene variants reflected by patients who showed delayed mental development. The seizures of *SCN1A* variant patients were predominantly focal seizures, followed by generalized tonic-clonic seizure (GTCS), while several patients exhibited comorbid myoclonus, spasms, absence seizures, and atonic seizures. *SCN2A* variant patients developed focal seizures, of whom three patients showed comorbid spasms. All *SCN8A* variant patients manifested focal seizures, of whom one patient had comorbid spasms; another patient exhibited comorbid spasms, absence seizures, and clonic seizures. Two *SCN3A* variant patients had focal seizures, of whom one showed comorbid spasms. *SCN9A, SCN11A*, and *SCN1B* variant patients experienced focal seizures and GTCS but did not experience any other types of epileptic seizures. With regard to epilepsy diagnosis, *SCN1A* variant patients were chiefly diagnosed with DS (72.7%, 40 of 55), and the remaining patients were diagnosed with unclassified epileptic encephalopathy (EE), GEFS+, complex febrile seizure (CFS), FS+, and West syndrome. Eight patients demonstrated comorbid ataxia, and two GEFS+ patients developed immune-related encephalitis during the course of the disease. Of 14 *SCN2A* variant patients, two were diagnosed with OS, one with West syndrome, one had epilepsy of infancy with migrating focal seizures (EIMFS), two had benign familial epilepsy, and eight had non-specific EE. One patient developed Todd's paralysis after convulsions and one patient acquired aphasia after convulsions; the latter was considered to be caused by speech-center involvement generated by the convulsions. One patient had autism spectrum disorder (ASD) and one showed bilateral hearing loss. Of nine *SCN8A* variant patients, one was diagnosed with West syndrome, one with Lennox–Gastaut syndrome (LGS), one with benign familial infantile epilepsy (BFIE), and six with non-specific EE. One patient manifested comorbid congenital joint dysplasia and two exhibited prolonged postpartum jaundice. Of the six *SCN9A* variant patients, four were diagnosed with GEFS+, one with CFS, one with non-specific EE, and one patient had comorbid cryptorchidism. Of the six *SCN1B* variant patients, three were diagnosed with GEFS+ and three with non-specific EE. Of the two *SCN3A* variant patients, one was diagnosed with non-specific focal seizures. This latter patient was diagnosed with ASD at 1 year and 9 months of age, had focal seizures at 2 years and 7 months of age, and exhibited spasms at 3 years of age. Of two *SCN11A* variant patients, one was diagnosed with EE and one with GEFS+ (Table 3).

**Table 3:** Clinical phenotypic characteristics of sodium channel gene variants.

	**Total (*n* = 94)**	**SCN1A (*n* = 55)**	**SCN2A (*n* = 14)**	**SCN8A (*n* = 9)**	**SCN9A (*n* = 6)**	**SCN1B (*n* = 6)**	**SCN3A (*n* = 2)**	**SCN11A (*n* = 2)**	**H**	***P***
Sex (male/female)	57/37	37/18	5/9	6/3	5/1	1/5	2/0	1/1		
Age at onset (months)	7.00 (4.5,10.2)	7.00 (5.0,9.0)	3.00 (0.1,23.0)	5.00 (3.5,7.5)	9.50 (7.3,18.0)	15.50 (7.5,22.0)	28.00 (25.0,31.0)	11.50 (10.0,13.0)	19.12	0.004
Age at follow-up (years)	6.00 (4.3,7.0)	6.00 (4.3,7.3)	5.12 (2.9,6.4)	5.00 (3.1,7.8)	7.92 (5.5,13.3)	8.50 (6.0,11.4)	6.96 (6.2,7.8)	12.50 (11.0,14.0)	16.321	0.012
Clustering	61	43	9	5	2	0	1	1		
Fever sensitivity	69	51	3	1	6	6	0	2		
Status epilepticus	33	26	3	2	0	1	0	1		
**Status of mental development**										
Normal	17	7	2	1	4	2	0	1		
Mild delay	7	5	0	0	1	0	1	0		
Moderate delay	22	14	3	1	0	3	0	1		
(Extremely) severe delay	48	29	9	7	1	1	1	0		
**Type of seizure**										
Focal	73	43	14	9	1	3	2	1		
GTCS	37	27	0	0	5	4	0	1		
Spasm	7	1	3	2	0	0	1	0		
Myoclonus	7	7	0	0	0	0	0	0		
Atypical absence	2	1	0	1	0	0	0	0		
Atonic	1	1	0	0	0	0	0	0		
Tonic	1	0	0	1	0	0	0	0		
**Diagnosis**										
DS	40	40	0	0	0	0	0	0		
WS	3	1	1	1	0	0	0	0		
OS	2	0	2	0	0	0	0	0		
LGS	1	0	0	1	0	0	0	0		
EIMFS	1	0	1	0	0	0	0	0		
Non-specific EE	25	5	8	6	1	3	1	1		
GEFS+	12	4	0	0	4	3	0	1		
BFE	3	0	2	1	0	0	0	0		
CFS	4	3	0	0	1	0	0	0		
FS+	2	2	0	0	0	0	0	0		
EP	1	0	0	0	0	0	1	0		
**Other accompanying symptoms**										
		Eight with limb ataxia and two with immune encephalitis	One with Todd's paralysis, one with acquired aphasia, one with autism spectrum disorder, and one with bilateral hearing loss	One with congenital joint dysplasia, two with prolonged postpartum jaundice	One with comorbid cryptorchidism	Unremarkable	One with autism spectrum disorder	Unremarkable		

### 3.3 Auxiliary examinations of patients with sodium channel gene variant-related epilepsy

All 94 pediatric patients underwent cranial magnetic resonance imaging (MRI) and the majority showed a normal head MRI (74.5%, 70 of 94). A minority of patients exhibited brain dysplasia or non-specific structural abnormalities. Eleven *SCN1A* variant patients produced an abnormal head MRI, of whom four had dilated cerebral hemispheric gyri, three had slightly intense signals in the frontal, parietal, and temporal white matter, two showed corpus callosum dysplasia, one had diminished hippocampal volume, and one manifested cerebral dysplasia. Seven *SCN2A* variant patients exhibited abnormal head MRIs, of whom five had a dilated extracerebral space and two possessed ependymal cysts. Five *SCN8A* variant patients produced abnormal head MRIs, of whom one showed cerebellar atrophy, two had a dilated extracerebral space, one had abnormal frontal cerebral blood vessel development, and one had slightly thicker and enhanced meninges in the right frontal lobe. Of the six *SCN9A* variant patients, one manifested brain white matter dysplasia in head MRIs and the remaining patients generated normal head MRIs. All *SCN1B, SCN3A*, and *SCN11A* patients showed normal MRIs. All pediatric patients underwent at least one video electroencephalography, and the majority showed abnormal electroencephalograms (76.6%, 72 of 94) that mainly indicated slow waves, focal discharge, generalized discharge, multifocal discharge, hypsarrhythmia, and burst suppression. Patients with *SCN1A, SCN2A, SCN8A*, and *SCN3A* variants were prone to background slowing. In addition, the first electroencephalograms were normal in 29 *SCN1A* variant patients, but electroencephalograms were abnormal upon 1-year follow-up in 18 patients (Table 4).

**Table 4:** Treatment and follow-up of epileptic patients with sodium channel gene variants.

	**Total (*n* = 94)**	**SCN1A (*n* = 55)**	**SCN2A (*n* = 14)**	**SCN8A (*n* = 9)**	**SCN9A (*n* = 6)**	**SCN1B (*n* = 6)**	**SCN3A (*n* = 2)**	**SCN11A (*n* = 2)**	***Z***	***P***
**ASMs (response to ASMs: controlled/reduction/failure)**										
VPA	17/33/27	5/26/18	1/2/4	1/2/4	6/0/0	2/1/1	0/2/0	2/0/0		
TPM	4/13/19	4/12/12	0/0/3	0/0/4	–	0/1/0	–	–		
LEV	9/1/42	5/1/24	2/0/11	0/0/5	1/0/1	0/0/1	–	1/0/0		
LCS	3/2/1	1/0/1	1/1/0	0/1/0	–	1/0/0	–	–		
OXC	5/3/9	0/0/4	3/1/2	2/2/1	–	–	0/0/1	0/0/1		
LTG	0/0/1	0/0/1	–	–	–	–	–	–		
CZP	1/2/3	1/2/3	–	–	–	–	–	–		
CLB	3/14/2	2/13/1	1/1/0	0/0/1	–	–	–	–		
KD	1/6/2	1/5/1	–	0/1/1	–	–	–	–		
ACTH	1/0/3	1/0/0	0/0/2	0/0/1	–	–	–	–		
PB	0/2/7	0/1/0	0/1/4	0/0/2	–	–	–	0/0/1		
DZP	2/0/0	1/0/0	–	–	–	1/0/0	–	–		
VGB	0/1/0	–	–	0/1/0	–	–	–	–		
CBZ	1/0/0	–	–	1/0/0	–	–	–	–		
**Response to ASMs**										
Controlled	39 (41.5%)	14 (25.4%)	8 (57.1%)	4 (44.4%)	6 (100%)	5 (83.3%)	0	2 (100%)	0.464	0.643
Reduction	43	35	4	2	0	0	2	0		
Failure	10	6	2	1	0	1	0	0		
Dead	5	2	1	2	0	0	0	0		

### 3.4 Treatment and follow-up results of patients with sodium channel gene variant-related epilepsy

**Figure 2:**
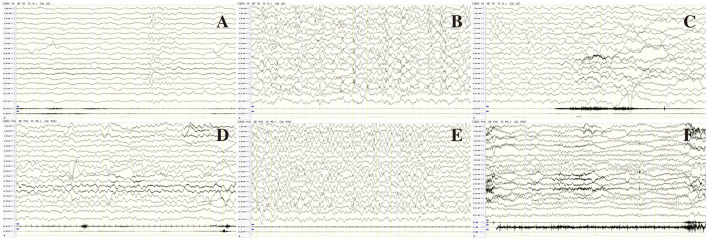
EEG of patient 80 in Table 1 before **(A–C)** and after **(D–F)** treatment of LCS. **(A)** Interictal EEG showed slow background, multiple focal discharges during wakefulness; **(B)** Atypical hypsarrhythmia during sleep; **(C)** Ictal EEG showed focal discharge associated with focal seizures. **(D)** Normal background activity during wakefulness after treatment with LCS; **(E)** Rare spikes during sleep; **(F)** Follow-up EEG showed normal background activity, rare spikes during wakefulness at age 1 year and 5 months.

**Figure 3:**
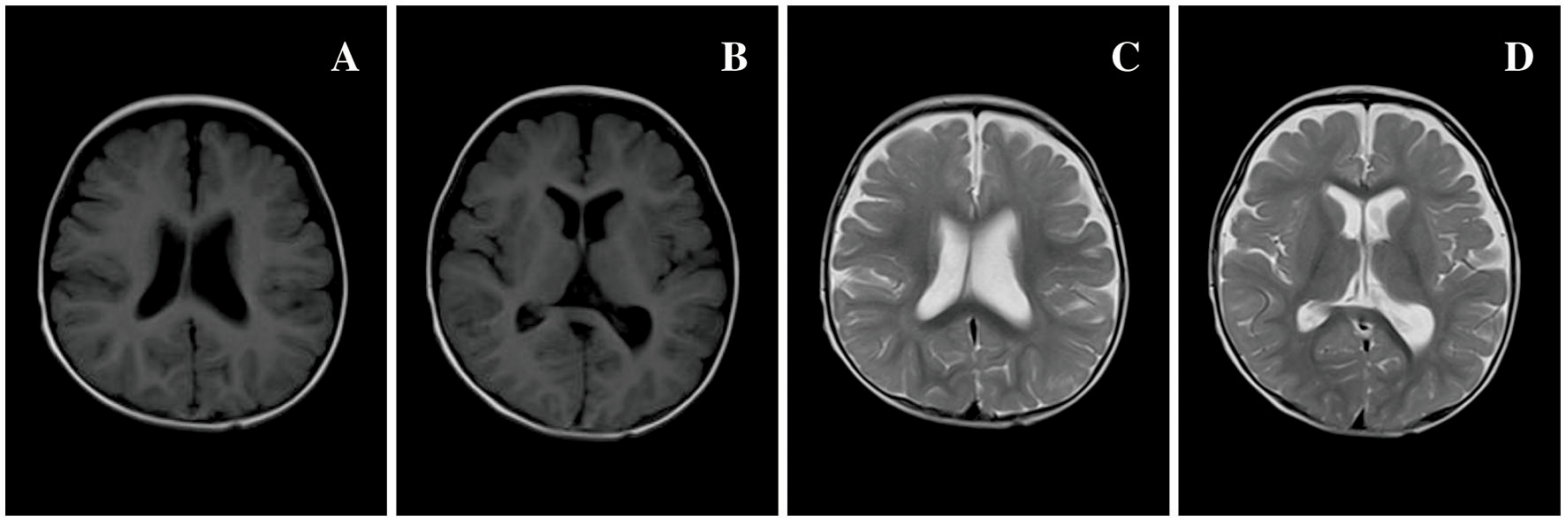
Brain MRI of 80 patients in Table 1 at 7 months of age. **(A, B)** T1 MRI and **(C, D)** T2 MRI: bilateral extracerebral spaces of frontal and temporal lobes widened, and the left ventricle was slightly dilated compared to the right side.

In the present study, one patient did not receive anticonvulsant drugs, while the remaining 93 received one or more anticonvulsant drugs. Patients were followed up to a median age of 6.0 (4.3, 7.0) years, and epilepsy symptoms were controlled (41.5%, 39 of 94) or reduced (45.7%, 43 of 94) in most patients. In *SCN1A* variant patients, anticonvulsants were effective in 89.1% (49 of 55) of patients, epilepsy was controlled in 25.5% (14 of 55), and the seizure frequency decreased in 63.9% (35 of 55). Drug efficacies in descending order were clobazam (CLB) at 93.8% (15 of 16), ketogenic diet (KD) at 85.7% (6 of 7), valproic acid (VPA) at 63.3% (31 of 49), topiramate (TPM) at 57.1% (16 of 28), diazepam (DZP) at 50.0% (3 of 6), lacosamide (LCS) at 50.0% (1 of 2), and levetiracetam (LEV) at 20.0% (6 of 30). Adrenocorticotropic hormone (ACTH) was effective in one patient, oxcarbazepine (OXC) did not show any improvement in four patients, and lamotrigine (LTG) was ineffective in one patient. In *SCN2A* variant patients, anticonvulsants were effective in 85.7% (12 of 14) of patients, epilepsy was controlled in 57.1% (8 of 14), and the seizure frequency decreased in 16.3% (4 of 14). Drug efficacies in descending order were LCS at 100% (2 of 2), CLB at 100% (2 of 2), OXC at 66.7% (4 of 6), VPA at 42.9% (3 of 7), and LEV at 15.4% (2 of 13). TPM was ineffective in three patients and ACTH was ineffective in two others. In total, six of the 14 patients with *SCN2A* variant epilepsy had early onset EE, of whom four received sodium channel blockers, seizures were controlled in 1 patient after OXC addition, and seizures decreased in one patient after OXC addition. Seizures diminished in one patient after LCS addition, and OXC addition was ineffective in one patient, but seizures completely resolved after LCS addition. This latter pediatric patient was an *SCN2A* variant (c.1285G>A)-related EIMFS patient who experienced convulsions on the first day after birth (Figures 2, 3). The neonate experienced over 50 seizures daily, and convulsions could not be controlled even after multiple anticonvulsants were administered. Seizures stopped when LCS was administered at 11 weeks, and convulsions were absent at one-and-a-half years of outpatient follow-up. In the present study, three patients with late-onset *SCN2A*-related epilepsy received OXC: OXC addition was ineffective in one patient, and seizures were completely controlled after OXC addition in the two others. In *SCN8A* variant patients, anticonvulsants were effective in 66.7% (six of nine) of patients, epilepsy was controlled in 25.5% (four of nine), and the seizure frequency declined in 22.2% (two of nine). Drug efficacies in descending order were carbamazepine (CBZ) at 100% (one of one), LCS at 100% (one of one), vigabatrin at 100% (one of one), OXC at 80% (four of five), KD at 50% (one of two), and VPA at 42.9% (three of seven). TPM was ineffective in all four patients treated, LEV was ineffective in all five patients treated, and ACTH was ineffective in the one patient treated. Epileptic seizures were controlled in six *SCN9A* patients (100%, six of six) and drug efficacies were 100% (six of six) with VPA and 50% with LEV (one of two). Epilepsy was controlled in 83.3% (five of six) of *SCN1B* variant patients. Three of four patients experienced efficacy with VPA (75%, three of four), and seizures were controlled in one patient treated with LCS. Seizures declined in two *SCN3A* patients and VPA was effective, whereas OXC was ineffective. Epilepsy symptoms were controlled in two *SCN11A* variant patients, in whom VPA and LEV were effective and where OXC was ineffective. Of all patients, five developed SUDEP, of whom two harbored an *SCN1A* variant; one patient was diagnosed with non-specific EE, one patient with DS, and two patients with a splicing variant. The age at death was 5 years of age in both patients, and one patient had a younger monozygotic twin sister who died of SE at 4 years of age. In *SCN1A* variant patients, one OS patient developed SUDEP at 7 months. Among the *SCN8A* variant patients, two developed SUDEP, one of whom with non-specific EE died at 3 years of age, and the other patient with West syndrome died at 5 years of age (Table 5).

**Table 5:** Auxiliary examinations of epileptic patients with sodium channel gene variants.

	**Total (*n* = 94)**	**SCN1A (*n* = 55)**	**SCN2A (*n* = 14)**	**SCN8A (*n* = 9)**	**SCN9A (*n* = 6)**	**SCN1B (*n* = 6)**	**SCN3A (*n* = 2)**	**SCN11A (*n* = 2)**
Total with abnormal brain MRI findings	24	11	7	5	1	0	0	0
**Patients with abnormal electroencephalogram**								
Total	72	44	12	8	3	3	1	1
Slow wave	44	28	10	5	0	0	1	0
Focal discharge	30	15	7	4	2	1	0	1
Generalized discharge	29	17	6	3	0	2	1	0
Multifocal discharge	15	8	4	2	1	0	0	0
Hypsarrhythmia	7	1	4	1	0	0	1	0
Burst suppression	3	0	2	1	0	0	0	0

### 3.5 Analysis of statistical results

We observed significant differences in the age at onset among the aforementioned seven subtypes of sodium channel gene variant-related epilepsy patients (*P* = 0.004, Table 3), revealing that age at onset may portend genetic heterogeneity. *SCN1A*/*SCN2A*/*SCN8A* variant-related epilepsy was generally manifested at an earlier age, while the *SCN3A*/*SCN9A*/*SCN1B* variant-related subtype showed a later age at onset. We noted no significant differences in treatment responses among the different subtypes (*P* = 0.643, Table 4).

## 4 Discussion

Ion channels constitute the material basis for electrical activity in neurons, and abnormalities in ion channel structure or function can result in electrophysiologic changes that cause abnormal epileptiform discharge. Most of the currently confirmed pathogenic mutations in epilepsy genes encode for ion channels or their auxiliary subunits; of these, gene variants in ion channels comprise the most common cause of genetic epilepsy and sodium channel-related epilepsy and reflect intense areas of research in recent years (11). Sodium channels include nine α subunits and four β subunits, with the *SCN1A, SCN2A, SCN3A, SCN8A, SCN9A, SCN11A*, and *SCN1B* encoding human Nav α1, α2, α3, α6, α7, α9, and β1, respectively. Different gene mutations then produce epileptic seizures of varying severity and types.

The *SCN1A* is located on chromosome 2q 24.3; it contains 31 exons and encodes the Nav1.1 α subunit. As our understanding of the *SCN1A* improves, we note an increasing number of reports describing *SCN1A* mutations. The diseases caused by various mutation sites and types are different and range from mild diseases such as GEFS+ and partial epilepsy with febrile seizures plus (PEFS+) to severe diseases such as DS (2). In our study, the 55 *SCN1A* variant patients encompassed those with relatively mild diseases (four with GEFS+, three with CFS, and two with FS+). With the exception of one patient with a frameshift variant, the remaining patients harbored missense variants. One study showed that the *SCN1A* mutation was localized to the pedigrees of 5–10% of GEFS+ patients. The GEFS+-related *SCN1A* mutation is principally concentrated in the intracellular domain of Nav1.1 (12), and almost all mutations are missense mutations, which is consistent with the results from the current study. A previous study indicated that truncation mutations and missense mutations resulted in the most severe clinical symptoms of all *SCN1A* mutations (13). We herein demonstrated that *de novo* splicing variants in the *SCN1A* were present in two patients and in the monozygotic younger sister of one patient, and that these patients died due to status epilepticus. Four patients who failed treatment were DS patients, of whom two harbored missense variants, one had a frameshift variant, and one had a duplication variant.; this, however, was different from previous studies and may have been due to our smaller sample size. A previous study showed that the most common gene mutations in *SCN1A*-related DS were frameshift mutations, missense mutations, and non-sense mutations; however, splice-site, duplication, and deletion mutations were rarely observed (14). Our study revealed that 40 *SCN1A* variant patients manifested DS, of whom 16 had missense variants, nine showed frameshift variants, eight had non-sense variants, six possessed splicing variants, and one exhibited a duplication variant; this was generally consistent with previous investigations. Analysis of the treatment responses in 55 patients revealed that CLB, KD, VPA, and TPM produced better efficacy in *SCN1A* variant-related epilepsy patients, LEV showed slight efficacy, and OXC and LTG were ineffective in controlling convulsions. Symptoms worsened in two of our GEFS+ patients as they experienced recurrent febrile seizures and were ultimately diagnosed with immune-related encephalitis. This suggested that the observed worsening of symptoms during treatment of patients with gene mutation-related epilepsy was not attributable solely to gene mutations, but that other factors such as immune factors and infection should be considered in order to adopt appropriate treatment.

Ever since the first case of *SCN2A* mutation-induced benign (familial) neonatal/infantile seizure was discovered and confirmed in 2001, the epilepsy spectrum as provoked by *SCN2A* mutations has continuously expanded to include OS syndrome, infantile spasms, EIMFS, DS, myoclonic-atonic epilepsy, LGS, GEFS+, and other unclassified severe EE (15, 16). Mild epilepsy is primarily related to inherited missense mutations and severe epilepsy is principally precipitated by *de novo* mutations, with the majority of patients showing delayed mental and motor development (17). In our study, 12 of 14 EE patients harbored *de novo* variants and manifested moderately severe mental developmental delay. Two children had benign familial epilepsy due to inherited variants, exhibited normal mental and motor development, and responded well to treatment, all of which was consistent with the literature (17). Studies revealed that patients with *SCN2A* mutation-related EE primarily showed neonatal or infantile onset, and over half showed a neonatal onset (8). In the present study, four EE patients experienced a neonatal onset and two had disease onset before 1–3 months of age. Six patients had a disease onset of 3 months or later, a situation completely different from that described in other reports. Studies showed that the *SCN2A* mutation generated two Nav1.2 functional changes: gain-of-function (GoF) and loss-of-function (LoF). Early-onset (age at onset < 3 months of age) Nav1.2 is mainly due to GoF and responds well to SCBs. Late-onset (onset ≥ 3 months of age) or non-epilepsy intellectual disability/autism spectrum disorder (ID/ASD) Nav1.2 is mainly due to LoF, responds poorly to SCBs, and may even augur for worsening seizures (8, 18). In our study, we applied sodium channel blockers to four of six patients with early-onset *SCN2A*-related epilepsy, and OXC and LCS were effective in two patients each. Although sodium channel blockers exhibit some efficacy in early-onset *SCN2A*-related epilepsy, OXC is not effective in all cases, and LCS and other sodium channel blockers can be used when OXC shows a poor response. We implemented OXC to treat three late-onset *SCN2A*-related epilepsy patients, and OXC was ineffective in one patient while seizures were completely controlled in the other two. It is obvious, then, that Nav1.2 electrophysiologic changes noted in *SCN2A* mutation-related EE are more complex. Studies have shown that many patients with epilepsy-related *SCN2A* mutations present with complex GoF or LoF characteristics, and some rare mutations share both characteristics (19, 20). Hence, it is difficult to apply a simple scheme with respect to classification. Additional experience in future diagnoses and treatments is certainly necessitated to establish more effective methods that can determine GoF or LoF characteristics, so as to produce suitable precision-therapy pharmaceuticals.

*SCN8A* encodes for the Nav1.6 α subunit and is highly expressed in the central nervous system. Since it was identified as an epilepsy-related gene in 2012, investigators have ascertained that *SCN8A* mutation-related epileptic seizures are diverse, as they can be manifested as systemic tonic-clonic seizures, spasms, absence, and focal seizures, and can also be present as epileptic syndromes that include BFIE or epileptic encephalopathies, such as Lennox-Gastaut syndrome and West syndrome. In addition to causing epileptic seizures, *SCN8A* mutations can affect speech, intelligence, and development in pediatric patients (21, 22). In our study, there were nine *SCN8A* variant-related epilepsy patients, of whom eight showed moderately severe mental and motor impairment. We observed diverse seizure forms but discerned that all patients showed focal seizures. Studies on the functionality of *SCN8A* mutations revealed that the electrophysiologic changes in the Nav1.6 channel included a persistent increase in Na+ current (INaP), complete channel inactivation, and steady-state rapid inactivation voltage-dependent depolarization transition (23). These three electrophysiologic changes are all GoF alterations of the Nav1.6 channel that increase neuronal excitation due to mutated channels, and correcting the GoF of this channel may therefore constitute a therapeutic target. A related study showed that high-dose sodium channel blockers such as carbamazepine, OXC, and phenytoin inhibited epileptic seizures in patients with the *SCN8A* mutation (24). In our study, six patients were treated with sodium channel blockers, and convulsions were controlled in one patient after CBZ was added; seizures were controlled in two patients and reduced in one patient; and treatment was ineffective in one patient after OXC treatment. In addition, although seizures decreased in one patient after OXC addition, they still occurred but diminished after LCS was added. This case shows that sodium channel blockers such as CBZ, OXC, and LCS are highly or partially effective in *SCN8A* variant-related epilepsy. In the present study, the seizure frequency did not decrease or increase in five patients after LEV was applied. VPA was used in seven patients and was effective in three patients and ineffective in four patients. This showed that LEV was ineffective in *SCN8A*-related epilepsy or that it may have even worsened epilepsy, while VPA exhibited some efficacy consistent with previous studies (25). One study depicted *SCN8A*-related EE as exhibiting a high mortality rate, reflecting the cause of death as SUDEP primarily. More than 10% of *SCN8A* mutation patients will develop SUDEP (26, 27), and two of nine patients (22%) died during epileptic seizures in our analysis.

*SCN9A* is located on chromosome 2q24.3, contains 27 exons, encodes for a 1977- amino-acid Nav1.7 α subunit, and was initially classified as a peripheral nervous system channel gene. It was subsequently reported that Nav1.7 is expressed in the cerebrum, particularly in the fetal hippocampus, showing that it plays a role in the central nervous system (28). Recent reports show that *SCN9A* mutations are mostly associated with febrile seizures and DS (29). One study showed that *SCN9A* mutations coexist with *SCN1A* mutations, that this mutation is a genetic modification factor in *SCN1A* mutation-related FS+, and that it may worsen the symptoms of mild FS+ (30). As another study showed that the *SCN9A* variant caused febrile seizures in families without the *SCN1A* variant (29, 31), it is possible that some *SCN9A* mutations may portray a modifying role in the presence of more potent mutations, or that it may itself induce mild epileptic seizures (32). There are very few extant reports on epilepsy caused by *SCN9A* mutations, and the pathogenesis of GEFS+ without an *SCN1A* mutation remains arcane. In our study, there were six *SCN9A* variant patients and all manifested fever sensitivity consistent with previous studies. All six patients had unreported missense variants and did not possess *SCN1A* variants. Of these, four patients were diagnosed with GEFS+, and one patient was diagnosed with CFS; we also reported a patient with non-specific EE. This latter patient began developing recurrent febrile convulsions at 5 months and received VPA as an anticonvulsant treatment. At 7 years and 6 months, seizures were controlled, although the patient exhibited severe developmental delay since infancy. In our study, seizures were controlled after one to two anticonvulsants were applied to six patients, and the response rate with VPA was 100% (six of six). This shows that *SCN9A* variant-related epilepsy predominantly consists of heterozygous missense variants. Most of the patients reflected fever sensitivity, prominently generalized seizures, and relatively mild symptoms; and anticonvulsants showed good efficacy in these patients.

*SCN1B* was the first sodium channel gene found to be associated with epilepsy, as Wallace et al. ascertained a point mutation in chromosome 19q13.1 in a GEFS+ pedigree in 1998 (33). Studies showed that heterozygous mutations in this gene were manifested as FS+ and others as non-EE (34), while patients with homozygous recessive mutations presented with DS-like EE symptoms and were at high risk of premature death (35). Compared with classical DS, the clinical symptoms of patients with homozygous *SCN1B* mutations are more consistent with early infantile developmental and epileptic encephalopathy (DEE), and the latter is more severe than DS (36). We found six *SCN1B* variant-related epilepsy patients. Three were diagnosed with GEFS+ and three with non-specific EE, and all six patients showed fever sensitivity; the three patients with non-specific EE had moderately mild DS-like symptoms. However, these six patients harbored heterozygous missense variants that were different from those described in previous studies (34, 35). Some investigators demonstrated that there were no differences in surface sodium channel β1 subunit expression in *SCN1B* mutant (p.Arg 85 Cys) cells compared with wild-type cells. However, the β1 subunit regulates Nav1.1 sodium ion current disappearance, revealing that this *SCN1B* mutation is a LoF mutation (34), thus providing a basis for the treatment of *SCN1B*-related epilepsy. With the exception of one patient in our study who did not take anticonvulsants regularly and who exhibited recurrent seizures, seizures were controlled in the other five patients. One patient did not self-administer any anticonvulsant drugs and convulsions did not occur after 6 years of age. Two patients received VPA monotherapy, one patient received diazepam tablets due to convulsion prophylaxis during fever, and another had a poor response to LEV—with seizures decreasing after the patient was switched to VPA and TPM, and controlled after adding LCS. Seizures were ultimately controlled in this patient after the sodium channel blocker LCS was applied, which was different from previous studies that showed that most *SCN1B* mutations were LoF mutations. Further evaluations of the specific biological mechanisms involved are therefore required.

The *SCN3A* is located on chromosome 2q24 and encodes for the Nav1.3 channel. Nav1.3 appears to be expressed at different sites at various times. For example, during embryonic and fetal development, Nav1.3 is principally expressed in the central nervous system, with its expression extremely low after birth. During infancy, Nav1.3 is gradually replaced by Nav1.1. *SCN3A* is also considered to be related to neurodevelopmental disorders and epilepsy, and *SCN3A* pathogenic mutations reflect many phenotypes. In mild cases, there is a mild cognitive disability, while in severe cases, there is a severe developmental delay due to cerebellar dysfunction (30). We herein noted two *SCN3A* variant patients, both with cognitive disabilities. The head MRI of these two patients was normal and there were no apparent structural abnormalities. These two *SCN3A* patients harbored missense variants. However, as our sample size was small, we only had a few *SCN3A* variant-induced epilepsy cases, and additional in-depth study is thus required.

The *SCN11A* codes for the sodium channel Nav1.9 is expressed in peripheral pan-sensing neurons and visceral afferent nerves and has been proven to be a threshold channel that is associated with peripheral neuropathy. There is no extant report of *SCN11A* and epilepsy. One study depicted the *SCN11A* variant as present in SUDEP patients, but it is unclear whether this variant causes epilepsy or SUDEP (37). We herein found two patients with epilepsy carrying gene variants in *SCN11A*: one patient was diagnosed with EE, with an unreported c.1646dupA frameshift variant in *SCN11A* from the patient's mother. The other patient was diagnosed with GEFS+ and had a reported c.2011T>C missense variant in *SCN11A* from the patient's father, who had a history of febrile seizures when he was young. Nav1.9 is specifically expressed in peripheral nociceptors, and animal models and clinical genetic studies showed that the channel protein plays an important role in inflammatory pain, neurogenic pain, and cold pain but its underlying mechanisms of action in epilepsy remain unelucidated. In our study, we enrolled two cases with *SCN11A* variants. However, more cases and further genetic functional verification are needed to confirm the relationship between *SCN11A* and epilepsy.

*SCN1A, SCN2A, SCN3A, SCN8A, SCN9A, SCN11A*, and *SCN1B* sodium channel gene mutations were intimately associated with epilepsy and displayed significant heterogeneity in pathogenesis and clinical symptoms. For example, *SCN1A* mutation-related epilepsy had a disease onset of several months, its clinical presentation was mainly DS, and few patients showed febrile seizures; while sodium channel blockers generally caused symptoms to worsen. *SCN2A* can cause early-onset encephalopathy within several days after birth or delayed epileptic encephalopathy after 3 months. A majority of seizures exhibited clustering and most sodium channel blockers provided favorable efficacy. Additionally, *SCN1A, SCN2A*, and S*CN8A* were associated with SUDEP. The treatment response to antiseizure medications (ASMs) varies depending on the type of Nav channel mutation. Therefore, it is of great significance to study the relationship between genotype, clinical phenotype, and efficacy of ASMs in children with epilepsy. Such studies should provide not only a reference for precise and individualized treatment of epilepsy but also a basis for genetic counseling and prenatal testing to maximize the benefits for children.

## Data availability statement

The original contributions presented in the study are included in the article/supplementary material, further inquiries can be directed to the corresponding author.

## Ethics statement

The studies involving humans were approved by the Ethics Committee of Hunan Children's Hospital. The studies were conducted in accordance with the local legislation and institutional requirements. Written informed consent for participation in this study was provided by the participants' legal guardians/next of kin. Written informed consent was obtained from the individual(s), and minor(s)' legal guardian/next of kin, for the publication of any potentially identifiable images or data included in this article.

## Author contributions

HF: Data curation, Formal analysis, Funding acquisition, Methodology, Supervision, Writing–original draft, Writing–review & editing. WH: Formal analysis, Writing–review & editing. QK: Investigation, Writing–review & editing. XK: Resources, Writing–review & editing. LWa: Writing–review & editing. XZ: Writing– review & editing. HL: Writing–review & editing. LY: Writing–review & editing. HY: Writing–review & editing. ZJ: Methodology, Writing–review & editing. LWu: Funding acquisition, Project administration, Resources, Writing–review & editing.
